# Comparing the Quality and Quantity of Extracted Deoxyribonucleic Acid (DNA) From Formalin-Fixed and Embalmed Tissues at Monthly Intervals Over Six Months

**DOI:** 10.7759/cureus.85238

**Published:** 2025-06-02

**Authors:** Nazrul Islam, Dinesh Kumar

**Affiliations:** 1 Department of Anatomy, All India Institute of Medical Sciences, Patna, IND; 2 Department of Anatomy, Maulana Azad Medical College, New Delhi, IND

**Keywords:** cadavers, dna extraction, dna quality, embalming tissues, formalin-fixed tissue

## Abstract

Background: Cadavers are the principal materials used for teaching and learning human anatomy. The dead bodies received from the Department of Anatomy are chemically treated (embalmed) to be learner-ready, arrest further decomposition, and preserve the body in a life-like state. The present study was designed to analyze this knowledge gap by extracting deoxyribonucleic acid (DNA) from different tissues of cadavers and to determine which tissue is more likely to provide DNA of suitable quality and quantity.

Materials and methods: This observational study was conducted at the Department of Anatomy, Maulana Azad Medical College (MAMC), New Delhi, India. It lasted one year, from January 2021 to January 2022. The Institutional Ethics Committee (IEC), MAMC, New Delhi, India, granted ethical approval under letter number F1/IEC/MAMC/82/10/2020/No.29 dated January 14, 2021.

Results: The mean concentration of all four tissues in all six bodies over six months was higher for formalin-fixed than embalmed muscle, but it was statistically not significant. It has been observed that the mean concentration of all tissues at 260/280 and 260/230 optical density was found to be statistically significant at a p value of less than 0.001. The preservatives used were formalin and embalming solution. While comparing data from all bodies, only muscles at 260/280 showed statistically significant results with a p value of 0.03.

Conclusion: The study concluded that for genomic studies, from the tissue preserved in embalming fluid among the four tissues studied, including liver, kidney, skin, and muscle, skin tissue is the best candidate, as purity is an essential requirement. The extracted DNA from the liver, kidney, and muscle tissues will require purification before being subjected to downstream procedures.

## Introduction

Cadavers are the principal teaching and learning materials of human anatomy. The dead bodies received from the Department of Anatomy are chemically treated (embalmed) to be learner-ready, arrest further decomposition, and preserve the body in a life-like state. Formalin is the principal chemical in the embalming fluid, which causes the cross-linking of proteins and binds with nuclear deoxyribonucleic acid (DNA). Due to these extensive bridging and chemical reactions, the DNA undergoes both chemical and physical stress, leading to its fragmentation. The embalming fluid contains not just formalin but a wide variety of chemicals like carbolic acid, methanol, thymol, salts, and buffers, which pose an additional challenge to nuclear DNA extraction [1].

Extensive studies are available on the extraction of DNA from formalin-fixed paraffin-embedded (FFPE) tissues. However, very few studies are on embalmed cadavers. FFPE, though available for genetic study, is a compromised tissue for nonpathological investigations [2-4].

Few studies, like the one by Wheeler et al. in 2016 on three cadavers 113, 290, and 814 days from the embalming, have compared the results of one cadaver to another and found that the DNA of an older cadaver (814 days) was more degraded than the DNA of the other two relatively new cadavers [5].

It is necessary to evaluate the quality of DNA extracted from preserved tissue samples and its impact on the reliability of downstream molecular applications in biomedical research.

The embalming fluid preserved cadavers can be used for DNA extraction, but the existing research data are primarily based on DNA extraction from FFPE. There is a paucity of literature on using cadavers as a source for DNA extraction. The present study was therefore designed to analyze this knowledge gap by extracting DNA from different tissues of cadavers and to determine which tissue is more likely to provide DNA of suitable quality and quantity.

## Materials and methods

Study design

This observational study was conducted at the Department of Anatomy, Maulana Azad Medical College (MAMC) in New Delhi, India. The study has been conducted for one year, i.e., from January 2021 to January 2022.

Study population

This study included six embalmed cadavers, involving three males and three females, available in the Department of Anatomy, MAMC, New Delhi, India. The inclusion criteria of participants included cadavers that were available in the department, in which the tissue DNA at day 0 was extractable. The exclusion criteria were cadavers with a history of liver, kidney, muscle, and skin diseases, anomalies, mutilation, and putrefaction. This was done by using available medical records and an inspection.

To avoid variables that might affect the DNA quality of the cadaver, a few points were considered, such as the age of the cadaver, which was all the same, ranging from 79 to 81. Also, a cold storage room was used to slow the degradation of DNA from death to embalming. Only those cadavers were selected whose cause of death was cardiovascular disease. As cardiovascular disease is the most common disease, cadavers were easily available.

Data collection

Quantitative and qualitative data of extracted DNA, such as tissue concentration (ng/µL) at different optical densities (ODs) from formalin-fixed and embalmed tissues at monthly intervals over six months, were obtained from different tissues involving the liver, kidney, skin, and muscles, respectively.

Study procedure

To select tissues, four types of tissues were dissected, involving skin over the lateral surface of the Gluteus region, Gluteus muscle, liver, and right kidney. These tissues were selected based on their position, i.e., deep (liver and kidney) and superficial regions (skin) of the body, to compare possible perfusion differences throughout the body.

All four tissues (skin, skeletal muscle, liver, and kidney) were harvested (first harvesting preembalming) on day 0. A smaller portion of approximately 0.5 cm^3^ of tissue was dissected from the harvested tissues and processed for DNA extraction. The remaining tissues were immersed and fixed in 20% formalin (20 times the volume of tissues) and kept at room temperature. Then, the cadaver was subjected to a standard embalming procedure.

The second harvesting of tissues (skin, skeletal muscle, liver, and kidney) was done after one month of embalming, and each tissue was kept in a separate jar filled with embalming fluid at room temperature. These tissues were used for future DNA extractions at one, two, three, four, five, and six months. The tissue was processed for DNA extraction from both formalin-fixed and embalmed fluid-fixed tissue at monthly intervals for six months.

The extracted DNA was made to run through a 0.8% Agarose gel and observed in the UV plate of the BIORAD Gel Documentation System. Further, the DNA samples were assessed using a nanodrop spectrophotometer for purity and concentration in the form of OD of DNA measured at 260/230 and 260/280 wavelengths.

Statistical analysis

The collected data were entered in MS Excel (Microsoft Corporation, Redmond, WA) and were analyzed using the Statistical Package for Social Sciences version 25 (IBM Corp., Armonk, NY). Quantitative data (DNA concentration) was expressed by mean and standard deviation, and a significant level of difference between the means was tested by Student’s t-test. One-way analysis of variance was applied to compare the quantitative values in different groups. F-test statistics and t-test statistics have been evaluated. A p value of less than 0.05 was considered statistically significant.

Ethical clearance

Ethical approval was granted by the Institutional Ethics Committee (IEC), MAMC, New Delhi, India under letter number F1/IEC/MAMC/82/10/2020/No.29 dated January 14, 2021.

## Results

**Figure 1:**
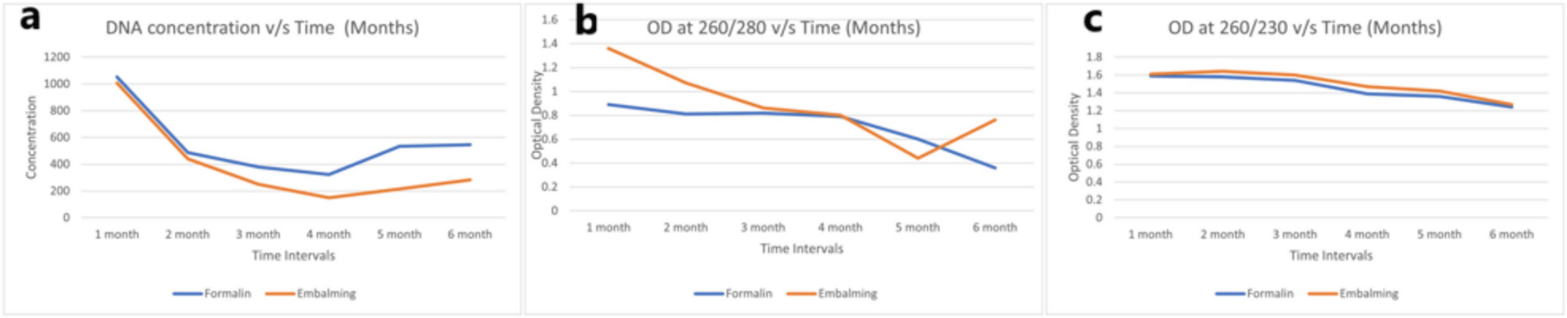
(a) DNA concentration, (b) OD (260/280), and (c) OD (260/230) vs. time for all female cadavers (A + B + C) as an average of all tissues (liver, kidney, skin, and muscle) OD: optical density

Figure 1 represents the concentration measured in OD (DNA concentration, at 260/280 OD, and 260/230) among all female cadavers in all tissues, including the liver, kidney, skin, and muscles. The value for both the formalin-fixed and embalmed tissue sharply declines in the first three months. Also, the value for embalming solution fixed tissue was higher than that of formalin-fixed tissue.

**Figure 2:**
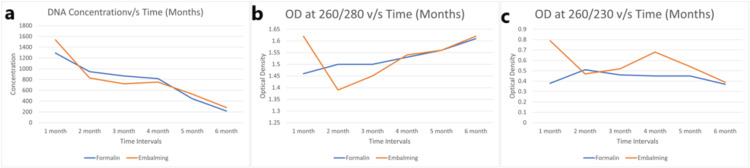
(a) Concentration, (b) OD (260/280), and (c) OD (260/230) vs. time for all male cadavers (1 + 2 + 3) as an average of all tissues (liver, kidney, skin, and muscle) OD: optical density

Figure 2 depicts the concentration measured in OD (DNA concentration, at 260/280 OD, and 260/230) among all male cadavers in all tissues, including the liver, kidney, skin, and muscles. It has been observed that after fixation of both the tissues of formalin-fixed and embalming, it shows a sharp decline in concentration in the first and second months; in the third and fourth months, it remains approximately constant; thereafter, in the fifth and sixth months, it again shows a brisk fall in concentration. The value for embalming solution fixed tissue was found to be better than formalin-fixed tissue at any given month, except in the second month.

**Figure 3:**
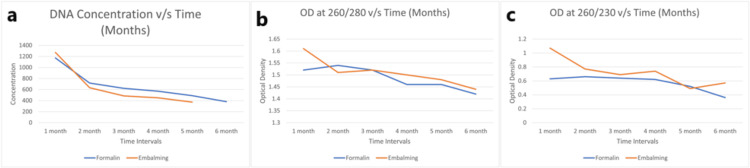
(a) Concentration, (b) OD (260/280), and (c) OD (260/230) vs. time for all bodies (A + B + C + 1 + 2 + 3) as an average of all tissues (liver, kidney, skin, and muscle)

Figure 3 shows the concentration measured in OD (DNA concentration, at 260/280 OD, and 260/230) among an average of all bodies, including (A + B + C + 1 + 2 + 3) cadavers in all tissues of the liver, kidney, skin, and muscles. It has been observed that, after fixation, the concentration goes down sharply in the first two months for both formalin-fixed and embalming fluid-fixed tissue; thereafter, it shows a gradual decline in the last four months. Also, the concentration of formalin-fixed tissue was found to be better than that of embalming fluid-fixed tissue.

Table 1 represents the mean concentration and OD of all tissues involving the liver, kidney, skin, and muscles. The mean concentration of all four tissues in all six bodies, over a period of six months, was higher for formalin-fixed than embalmed muscle, but it was not statistically significant. It has been observed that the mean concentration of all tissues at 260/280 and 260/230 OD was found to be statistically significant at a p value of less than 0.001.

**Table 1:** Mean concentration and optical density for all tissues One-way ANOVA test was used to obtain a p value A p value of less than 0.05 was considered significant ANOVA: analysis of variance

Parameter	Preservative	n	Mean	Standard deviation	F-statistic value	p value
All tissue concentration	Normal	6	1,035.04	608.44	1.88	0.159
	Formalin	36	657.19	528.29		
	Embalming	36	580.29	524.34		
	Total	78	650.76	538.25		
All tissue 260/280	Normal	6	1.78	0.07	9.55	<0.001
	Formalin	36	1.49	0.16		
	Embalming	36	1.52	0.15		
	Total	78	1.52	0.16		
All tissue 260/230	Normal	6	1.15	0.41	8.65	<0.001
	Formalin	36	0.57	0.28		
	Embalming	36	0.72	0.35		
	Total	78	0.69	0.35		

The mean concentration, OD 260/280, and OD 260/230 of all the tissues decreased over six months, and this was statistically significant at a p value of less than 0.05. Table 2 depicts the mean concentration and OD of all tissues for six months.

**Table 2:** Mean concentration and optical density for all tissues over six months A one-way ANOVA test was used to obtain the p value A p value of less than 0.05 was considered significant ANOVA: analysis of variance

Parameter	Months	n	Mean	Standard deviation	F-statistic value	p value
All tissue concentration	0	6	1,035.04	608.44	5.28	<0.001
	1	12	1,211.12	655.41		
	2	12	691.62	519.92		
	3	12	555.06	420.83		
	4	12	491.06	426.65		
	5	12	430.83	343.73		
	6	12	332.77	238.21		
	Total	78	650.76	538.25		
All tissue 260/280	0	6	1.77	0.07	4.53	<0.001
	1	12	1.58	0.12		
	2	12	1.53	0.15		
	3	12	1.52	0.13		
	4	12	1.48	0.15		
	5	12	1.48	0.12		
	6	12	1.42	0.21		
	Total	78	1.52	0.16		
All tissue 260/230	0	6	1.15	0.41	4.99	<0.001
	1	12	0.89	0.37		
	2	12	0.70	0.33		
	3	12	0.66	0.31		
	4	12	0.67	0.31		
	5	12	0.51	0.12		
	6	12	0.45	0.29		
	Total	78	0.69	0.35		

Table 3 compares all bodies over six-month periods. The preservatives used were formalin and embalming fluid. While comparing data from all bodies, only muscles at 260/280 showed statistically significant results, with a p value of 0.03.

**Table 3:** Comparison of data of all bodies over six-month periods An independent t-test was used to obtain the p value A p value of less than 0.05 was considered significant

Parameter	Preservative	n	Mean	Standard deviation	t-value	p value
Liver concentration	Formalin	36	1,039.88	862.47	-0.67	0.503
	Embalming	36	896.90	939.06		
Liver 260/280	Formalin	36	1.55	0.13	-0.56	0.640
	Embalming	36	1.53	0.17		
Liver 260/230	Formalin	36	0.73	0.30	1.47	0.149
	Embalming	36	0.88	0.53		
Kidney concentration	Formalin	36	714.81	755.01	-0.11	0.911
	Embalming	36	695.06	733.42		
Kidney 260/280	Formalin	36	1.53	0.19	-0.48	0.666
	Embalming	36	1.51	0.16		
Kidney 260/230	Formalin	35	0.70	0.36	1.04	0.327
	Embalming	36	0.80	0.45		
Skin concentration	Formalin	36	314.02	319.36	0.04	0.963
	Embalming	36	317.94	383.40		
Skin 260/280	Formalin	36	1.55	0.22	0.38	0.623
	Embalming	36	1.57	0.22		
Skin 260/230	Formalin	35	0.52	0.33	1.52	0.131
	Embalming	36	0.66	0.44		
Muscle concentration	Formalin	36	560.05	499.99	-1.43	0.156
	Embalming	36	411.26	370.82		
Muscle 260/280	Formalin	36	1.33	0.22	2.22	0.038
	Embalming	36	1.44	0.20		
Muscle 260/230	Formalin	32	0.43	0.32	1.94	0.076
	Embalming	35	0.57	0.29		
All tissue concentration	Formalin	36	657.19	528.29	-0.62	0.537
	Embalming	36	580.29	524.34		
All tissue 260/280	Formalin	36	1.49	0.16	0.82	0.513
	Embalming	36	1.52	0.15		
All tissue 260/230	Formalin	36	0.57	0.28	2.0	0.054
	Embalming	36	0.72	0.35		

## Discussion

Cadaver is the principal study material in the Anatomy Department. To make it study-ready for routine academic purposes, it has to undergo chemical processing, i.e., embalming, to stop its further decomposition and preserve it in a life-like condition. The standard embalming fluid is composed of formaldehyde, methanol, carbolic acid, glycerine, oxalates, and buffers. In modern evidence-based medical practice, archival preserved cadaveric tissue constitutes an invaluable resource for histological examination, molecular diagnostic procedures, and DNA typing analysis in forensic investigations [6].

In the present study, four tissues (two superficial, i.e., skin and muscle, and two deep, i.e., liver and kidney) were selected from six cadavers to investigate the concentration and purity of extracted DNA and its status over six months. In their study on autopsied bodies without chemical preservation, DNA was effectively isolated from the brain, liver, spleen, lymph nodes, kidney, psoas muscle, and prostate gland by Atmadja et al. using the Bar and Kirby techniques and spectrophotometric measurement. They showed that the liver, kidney, lymph nodes, and spleen contributed more DNA than the other organs. Most of the samples had high-molecular-weight DNA with varying degrees of degradation, according to their agarose gel electrophoresis [7].

In the present study, liver tissue had the maximum concentration of genomic DNA (1039 ng/µL for formalin-fixed and 896 ng/µL for embalming fluid-fixed, whereas skin had the least (314 ng/µL for formalin-fixed and 317 ng/µL for embalming fluid-fixed skin). Tissue-specific structural complexity explains the variation in DNA quality and purity. There are very few fibrous cells among the delicate membrane cells that make up the liver, kidney, and brain tissues. In contrast, keratin and other fibrous cells make up the stratified tissue that makes up the skin. Numerous proteins within the cell make up muscle tissue. Lipids make up the majority of adipose tissue, which causes cell volume to increase and cell number to decrease. Furthermore, because lipids are insoluble in water, extraction is hampered, and fewer nuclei are lysed [8].

According to Campos and Gilbert, DNA deterioration is caused by extremely acidic fixatives, extended storage times, and storage in warm conditions for extended fixation, particularly for more than 12-24 hours [9]. The present study confirms that the quality and quantity of extracted DNA in postmortem preserved tissues at different time intervals deteriorate at room temperature. Another study examined the muscle, liver, kidney, brain, adipose, and skin tissues of pigs. According to spectrophotometric measurements, DNA concentrations from various organs varied, with the liver and adipose tissues exhibiting the highest and lowest quantities, respectively [10].

Age and cause of death, the amount of time between death and the embalming procedure, and the patency of the peripheral blood arteries all have an impact on the quality of embalmed tissue [11]. Formalin-based embalming fluid cadavers were used in a study by Gerhard et al. to extract slices of the liver, skin, cardiac atrium and ventricle, and skeletal muscle for DNA analysis for exome sequencing. The first two tissues with a comparatively large DNA concentration were the liver and the heart [12].

The limitation of the study involved that there were differences noted in the male and female cadavers; however, the small sample size was not sufficient to comment on it and required expansion of the sample size.

## Conclusions

The study concluded that for genomic studies, from the tissue preserved in embalming fluid among the four tissues studied, including liver, kidney, skin, and muscle, skin tissue is the best candidate, compared to where purity is an essential requirement. The extracted DNA from the liver, kidney, and muscle tissues will require purification before being subjected to downstream procedures. However, muscle tissue should not be preferred in comparison to other studied tissues, where purity is a matter of concern. The concentration of extracted DNA is maximum for the liver and least in the skin for both formalin and embalming fluid-preserved tissues. The purity of DNA is better in embalming fluid-preserved cadavers in skin.

Routine DNA extraction shall be prescribed for all cadavers received in the Anatomy Departments for correlating with anomalies encountered in the course of dissections.

## References

[1] Brenner E (2014). Human body preservation - old and new techniques. J Anat.

[2] Yi QQ, Yang R, Shi JF, Zeng NY, Liang DY, Sha S, Chang Q (2020). Effect of preservation time of formalin-fixed paraffin-embedded tissues on extractable DNA and RNA quantity. J Int Med Res.

[3] Weiss AT, Delcour NM, Meyer A, Klopfleisch R (2011). Efficient and cost-effective extraction of genomic DNA from formalin-fixed and paraffin-embedded tissues. Vet Pathol.

[4] Paireder S, Werner B, Bailer J, Werther W, Schmid E, Patzak B, Cichna-Markl M (2013). Comparison of protocols for DNA extraction from long-term preserved formalin fixed tissues. Anal Biochem.

[5] Wheeler A, Czado N, Gangitano D, Turnbough M, Hughes-Stamm S (2017). Comparison of DNA yield and STR success rates from different tissues in embalmed bodies. Int J Legal Med.

[6] Duval K, Aubin RA, Elliott J (2010). Optimized manual and automated recovery of amplifiable DNA from tissues preserved in buffered formalin and alcohol-based fixative. Forensic Sci Int Genet.

[7] Atmadja DS, Tatsuno Y, Ueno Y, Nishimura A (1995). The effect of extraction methods. The kind of organ samples and the examination delay on the DNA yields and typing. Kobe J Med Sci.

[8] Mansano CFM, Pereira MM, Macente BI, Makino LC, Jacintho APP, Nakaghi LSO, De Stéfani MV (2016). Different protein levels in the diet of bullfrog tadpoles (Lithobates catesbeianus) and its effects on the liver tissue. Braz Vet Res.

[9] Campos PF, Gilbert TM (2012). DNA extraction from formalin-fixed material. Methods Mol Biol.

[10] Biase FH, Franco MM, Goulart LR, Antunes RC (2002). Protocol for extraction of genomic DNA from swine solid tissues. Genet Mol Biol.

[11] Nicholson HD, Samalia L, Gould M, Hurst PR, Woodroffe M (2005). A comparison of different embalming fluids on the quality of histological preservation in human cadavers. Eur J Morphol.

[12] Gerhard GS, Jin Q, Paynton BV, Popoff SN (2016). The Anatomy to Genomics (ATG) Start Genetics medical school initiative: incorporating exome sequencing data from cadavers used for Anatomy instruction into the first year curriculum. BMC Med Genomics.

